# Relationships between Expenditure of Regional Governments and Suicide Mortalities Caused by Six Major Motives in Japan

**DOI:** 10.3390/ijerph19010084

**Published:** 2021-12-22

**Authors:** Toshiki Hasegawa, Kouji Fukuyama, Motohiro Okada

**Affiliations:** Department of Neuropsychiatry, Division of Neuroscience, Graduate School of Medicine, Tsu 514-8507, Japan; t-hasegawa@med.mie-u.ac.jp (T.H.); k-fukuyama@clin.medic.mie-u.ac.jp (K.F.)

**Keywords:** suicide mortality, Japan, governmental expenditure, welfare, education

## Abstract

Suicide mortality in Japan reduced in the period of 2009–2018. A number of studies identified the impact of financial governmental support for social welfare systems on suicide mortality; however, the detailed effects of specific regional policies, designed according to regional cultural, economic, education and welfare situations, on suicide mortality remain to be clarified. Therefore, the present study analyses the associations between the regional governmental expenditure of six major divisions, “public health”, “public works”, “police”, “ambulance/fire services”, “welfare” and “education”, and suicide mortalities caused by six major suicidal motives, related to “family”, “health”, “economy”, “employment”, “romance” and “school”, across the 47 prefectures in Japan during the period of 2009–2018, using fixed-effect analysis of hierarchal linear regression with robust standard error. The expenditure of “public works” displayed a positive relationship with suicide mortality of females caused by family-related motives but was not related to other suicide mortalities, whereas the expenditures in “public health”, “police”, “ambulance/fire services”, “welfare” and “education” contributed to a reduction in suicide mortality, at least in some statistical indicators. The expenditures of both “ambulance/fire” and “education” were predominantly effective among the six major divisions of regional governmental expenditure in reducing suicide mortalities. In the education subdivisions, the expenditure of “kindergarten” was related to a reduction in suicide mortalities caused by a wide spectrum of motives. The amount of expenditure of welfare indicated the limited possibility of facilitating a reduction in suicide mortalities caused by only motives associated with economy or employment. However, in the welfare subdivisions, the expenditure of “child welfare” and “social welfare” was effective in reducing suicide mortalities, but the expenditure of “elderly welfare” was unexpectedly related to an increase in suicide mortalities. These results suggest that most Japanese people are struggling to bring up children even in the situation of an increasing elderly population with a decreasing birth rate. Therefore, it is important to enhance the investment welfare policy for the future to improve the childcare environment. Although the issue of an increasing elderly population and a decreasing birth rate in Japan has not yet improved, the obtained results suggest that evidence-based welfare expenditure redistributions of prefectures and municipalities could improve Japanese society and welfare systems.

## 1. Introduction

Prevention of suicide continues to be the most important worldwide public health issue [1,2,3,4,5]. Over the past three decades, Japanese suicide mortality has experienced drastic fluctuations. In fact, in the early 1990s, the suicide mortality of Japanese males (17–18 per 100,000 population) was not as high as compared to that of males in European countries [6,7,8,9]. However, following the collapse of the asset bubble in 1991 and the Asian economic crisis in 1997, Japanese suicide mortality drastically rose to more than 30,000 deaths in 1998 (at maximum, 40.1 deaths per 100,000 males in 2003) [10,11,12]. The Japanese people had to endure this public health crisis for 10 years until 2009, from which point onward, suicide mortality began to lower. Several studies have reported the hypothesis that the overlapping of socioeconomic stagnation during the “lost decades” induced by the collapse of the asset bubble and the additional economic crisis induced by the Asian economic crisis played an important role in the mechanisms of this tragedy of the drastic and persistent increase in suicide mortality in Japan [6,10,12,13,14]. In spite of these efforts, the actual mechanisms of the decrease in suicide mortality in Japan, which has continued for more than a decade since 2009, remain to be clarified.

Macroeconomic status and unemployment rates in Japan were deteriorated by the 2008 global financial crisis, similar to the situation in other Western and Asian countries; however, suicide mortality in Japan began to decrease in 2009 [15,16]. Unlike in Japan, although employment conditions and other macroeconomic situations in the U.K. began to recover in 2011, suicide mortality did not decrease even after economic improvement in the U.K. [17,18,19]. The discrepancy associated with kinetics of suicide mortalities between Japan and the U.K. is probably caused by the different politics in both countries. In 2010 and 2012, the coalition government in the U.K. implemented policies (austerity and welfare reform) to improve public deficits that had drastically increased due to the 2008 global financial crisis [20]. Indeed, during austerity and welfare reform, the severe cuts to regional governmental expenditures adversely affected various regional welfare services to households with disabilities and lower incomes [21]. Additionally, an increase in suicide mortalities was also observed in several other countries, such as Spain, Portugal, Greece and Ireland, which implemented austerity after the 2008 global financial crisis, resembling the U.K. [20,22,23,24,25,26,27,28,29]; however, the enhancement of suicide prevention programmes and the social welfare system in Denmark prevented an increase in suicide mortality induced by the 2008 global financial crisis [30].

Although national governmental expenditures in Japan did not significantly increase during the period of 2009–2018, the Japanese government provided prefectures/municipalities with the “Emergency Fund to Enhance Community-Based Suicide Countermeasure” to encourage regional suicide prevention programmes in 2009 [31,32,33]. The trends among policies and suicide mortalities in Denmark, Japan and the U.K. after the 2008 global financial crisis suggest that not only macroeconomic indicators but also governmental financial expenditures play important roles in suicide mortalities [7,16,17,34,35]. That is, increasing and decreasing financial expenditures probably contribute to reductions and increases in suicide mortality, respectively. Indeed, it has been well established that welfare generosity and governmental financial support for the enhancement of social security systems contribute to a decrease in suicide mortalities in several countries [19,29,36,37,38,39]. In the USA, increased financial contribution to Medicaid is considered to facilitate access to healthcare, resulting in reduced suicide mortality [40]. An increase in states participating in supplemental nutrition assistance programmes (supports individuals with low/no income) to purchase food has also been observed to be related to a decrease in suicide mortality in the USA [19]. In Italy, vocational rehabilitation programmes have contributed to decreasing the suicide mortality of working-age males due to the suppression of social isolation by unemployment, supporting individuals to continue to integrate into society [38,39]. In South Korea, increasing governmental support for social welfare facilities for the elderly decreased suicide mortality in regions with a higher proportion of elderly residents at a high risk of suicide [36]. Furthermore, a recent study reported that the encouragements of financial expenditures to public health, welfare, education and employment may have contributed to the improvement in suicide mortality in India [41].

We previously reported that the Emergency Fund to Enhance Community-Based Suicide Countermeasure contributed to a reduction in suicide mortalities in predominantly elderly males in Japan, but its effect on females and the younger population was limited [7,16,34,42]. Susceptibility analyses of suicide prevention programmes revealed that several factors associated with the life cycles of the individual, such as gender, age, work–life balance and family–life balance, had great impacts [34,42,43]. Indeed, prefectural enlightenment and intervention programmes contributed to a reduction in suicide mortality of males, whereas female suicide mortality was decreased by municipal development of leaders/listeners programmes but not by any prefectural programme [7]. Furthermore, Japanese regional suicide prevention programmes facilitated a reduction in the suicide mortality of the elderly population but had less marked effects on the suicide mortality of the younger population [16]. Although regional government expenditure is composed of essential expenditure for maintaining regional social and welfare systems and limited expenditures for their own operating politics, the features of regional governmental expenditures during the period of 2009–2018 may have contributed to the prevention of suicide [35]. However, little attention has been paid to how specific regional policies, designed according to regional, cultural, economic and social welfare situations, affect suicide mortality caused by the aforementioned suicidal motives in Japan. Therefore, to clarify the more detailed impacts of regional governmental policies on suicide mortality, the present study analysed the complicated effects of the regional governmental expenditure of six major divisions, namely, “public health”, “public works”, “police”, “ambulance/fire services”, “welfare” and “education”, on and suicide mortalities caused by six major suicidal motives, problems related to “family”, “health”, “economy”, “employment”, “romance” and “school”, across the 47 prefectures in Japan from 2009 to 2018 using fixed-effect analysis of hierarchal linear regression with robust standard error.

## 2. Materials and Methods

### 2.1. Dependent Variables

The numbers of suicide victims in 47 prefectures in Japan from 2009 to 2018 were obtained from the Basic Data on Suicide in the Region (BDSR) in the national database in the MHLW [9]. BDSR makes available the numbers of annual suicide victims disaggregated by six major suicide motives: family-, health-, economy-, employment-, romance- and school-related problems [9]. Annual prefectural suicide mortalities caused by suicidal motives are calculated by dividing numbers of suicide victims per prefecture by the prefectural population (denominator) of the same years. The prefectural population was obtained from the Regional Statistics Database: System of Social and Demographic Statistics of the Statistics Bureau of the Ministry of Internal Affairs and Communications (SBMIAC) [44]. To eliminate small prefectural population artifacts, prefectural suicide mortality was calculated by using the empirical Bayes standardised mobile ratio method by using the empirical Bayes estimator for the Poisson/gamma model (v2.1) (National Institute of Public Health, Wako, Japan) (https://www.niph.go.jp/soshiki/gijutsu/download/ebpoig/index_j.html, accessed on 14 August 2021) [45]. Annual standardised death rates for the suicide mortality (SDR) of males, females and both genders (males plus females) were calculated on the basis of the Japanese age-dependent population composition in 2009 for males and females.

BDSR published the number of suicide victims caused by suicidal motives that were collected by the police. The police explore personal characteristics based on background factors of each suicide victim in order to delve deeply into the heart of the suicidal motive. The investigation derives the possible motives for suicide, based on the evidence, suicide note and/or other several documentations, such as medical records, clinical records and testimony of surviving family. Finally, police identify the suicidal motive of each suicide victim from several motives compared with previous compiled lists of suicide motives [9].

### 2.2. Independent Variables

Regional financial expenditure was obtained from the Survey of Local Public Finance Settlement (SLPFS) in SBMIAC [46,47]. The regional governmental expenditures are composed of the expenditure of six divisions, namely, “public health”, “public works”, “police service”, “ambulance/fire services”, “welfare” and “education” [35,46,47]. Furthermore, SLPFS makes available the expenditures per capita of the subdivisions of education and welfare [35,46,47]. The education expenditure is disaggregated by six subdivisions, “social education”, “elementary school education”, “junior high school education”, “senior high school education”, “special school education” and “kindergarten school education”35,46,47]. The welfare expenditure is disaggregated by four subdivisions, “social welfare”, “elderly welfare”, “child welfare” and “livelihood welfare” [35,46,47]. 

The present study adopted two types of independent valuables: the ratio of the expenditure (%) in Model-1 and the expenditure per capita (JPN/individual) in Models-2, -3 and -4 of six divisions in each regional government, published in SLPFS [35,46,47]. The ratio of expenditures of the six divisions in SLPFS was calculated by dividing their expenditures by the total amount of expenditure in each prefecture (denominator) [35]. The expenditures per capita of the six divisions in SLPFS were calculated by dividing their expenditures by the total population in each prefecture (denominator) [35]. Expenditures per capita of the education subdivisions, namely, special, kindergarten, elementary, junior high and senior high schools, were calculated by dividing their expenditures by the student numbers of each school category of the prefectures [35,46,47]. Expenditures per capita of the welfare subdivisions, namely, elderly, child, livelihood and social welfare, were calculated by dividing their expenditures by populations of individuals older than 65 years old, younger than 18 years old, assisted by livelihood protection and total population in each prefecture, respectively [35,46,47].

An explanation of the targets, objects and breakdown of each expenditure is given in Supplementary Table S1.

### 2.3. Statistical Analysis

In general, panel data application analyses the impact of the main parameters of interest on suicide mortality, followed by further analysis of the addition of various risk or protection predictors that are expected to affect suicide mortality in the literature leading to the identification of the overall background of suicide. The present study analysed the relationship between regional governmental expenditures and suicide mortality. Contrary to the general panel data analysis strategy, we investigated the effective divisions of regional governmental expenditures via subdividing the governmental expenditures [35]. Fixed-effects models can control for unobserved time-invariant factors, such as culture, climate, economic and educational situations etc., that may affect regional governmental expenditures, resulting in an effect on regional suicide mortality in each year. Prefectural fixed-effects cannot correspond changes over time, and year fixed-effects cannot fully account for state-specific trends in predictors. Therefore, according to panel data applications [35,48,49], the regression model of the four-step analysis was as follows: suicide mortality = γ_00_ + $\sum_{i=1}^{n}($(γ_10*n_* (expenditure) ij + γ_10*n + 1_* (centred expenditure) _j_* (expenditure) ij + u_0j_ + r_ij_ (residual), where (expenditure) is the regional governmental expenditures of divisions or subdivisions using HLM7 (Scientific Software International, Skokie, IL, USA). Furthermore, the present study adopted the robust standard errors clustered by prefecture to account for the issue that, similar to other forms of regression analyses, fixed-effects models can be subject to heteroscedasticity and autocorrelation [15,50]. Hierarchical data were applied to a hierarchical linear regression analysis when the intraclass correlation coefficient value was greater than 0.1 or the design effect value was greater than 2. This criterion was also applied to the theoretical justification regarding the inclusion of the random effect in the equations. There were no missing independent or dependent values in this study; thus, the sample size was *n* = 470, with 47 prefectures (*n* = 47) and 10 years (between 2009 and 2018, *n* = 10).

In the first step, the financial expenditure ratios of prefectures/municipalities and suicide mortality caused by suicidal motives were analysed by a hierarchical linear regression model with robust standard errors clustered at the prefectural level using HLM7 (Model-1) [15,35]. In the second step, the regional financial expenditure per capita of six major divisions and suicide mortality caused by suicidal motives were analysed (Model-2). In the third step, the regional financial expenditure per capita of divisions with education subdivisions and suicide mortality caused by suicidal motives were analysed (Model-3). In the final step, the regional financial expenditure per capita of divisions with subdivisions of education and welfare and suicide mortality caused by suicidal motives were analysed (Model-4).

The trends of regional governmental expenditures per capita of welfare subdivisions, social welfare, elderly welfare, child welfare and livelihood welfare in Japan in 2009–2018 were analysed by a linear mixed model using BellCurve for Excel v.3.2 (Social Survey Research Information Co., Ltd., Tokyo, Japan) [51,52,53]. When the data did not violate the assumption of sphericity (*p* > 0.05), the F value of the linear mixed model was analysed by sphericity assumed degrees of freedom, whereas when the assumption of sphericity was violated (*p* < 0.05), the F value was analysed by Chi–Muller-corrected degrees of freedom. Finally, data were analysed using Tukey’s post hoc test when the F values were significant [54,55].

## 3. Results

### 3.1. Relationship between Total Amount of Regional Financial Expenditure of Six Divisions and Suicide Mortalities Caused by Six Major Motives (Model-1)

**Figure 1:**
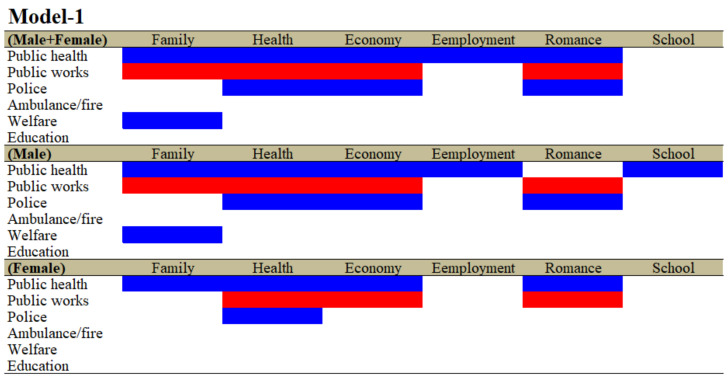
Relationship between total amount of financial expenditures of six major divisions (public health, public works, police, ambulance/fire, welfare and education) in prefectures/municipalities and suicide mortalities caused by six major motives (family-, health-, economy-, employment-, romance- and school-related problems) for both genders (males + females), males and females between 2009 and 2018 using fixed- or random-effect analyses of hierarchal linear regression with robust standard error. Blue and red columns denote significant factors for decreasing and increasing suicide mortalities, respectively. The detailed statistical values are presented in Supplementary Tables S2–S4.

The hierarchical linear regression model detected positive and negative relationships between the total amount of the financial expenditure of the six major divisions of prefectures/municipalities (public health, public works, police, ambulance/fire, welfare and education) and suicide mortalities caused by the six major suicide motives (family, health, economy, employment, romance and school problems) for both genders (males plus females, hereafter males + females), males and females (Figure 1 and Supplementary Tables S2–S4).

Suicide mortalities with family-related motives for both males + females and males were decreased by the total amount of expenditure of public health and welfare but were increased by the total amount of expenditure of public works (Figure 1 and Supplementary Tables S2 and S3). Suicide mortalities caused by family problems for females were decreased by the total amount of public health expenditures (Figure 1 and Supplementary Table S4). Suicide mortalities with health-related motives for males + females, males and females were decreased by the total amount of public health and police expenditures but were increased by the total amount of expenditure of public works (Figure 1 and Supplementary Tables S2–S4). Suicide mortalities with economy-related motives for males + females and males were decreased by the total amount of expenditures for public health and police but were increased by the total amount of expenditure of public works (Figure 1 and Supplementary Tables S2 and S3). Suicide mortalities with economy-related motives for females were decreased by the total amount of public health expenditures but were increased by the total amount of expenditure of public works (Figure 1 and Supplementary Table S4). Suicide mortalities with employment-related motives for males + females and males were decreased by the total amount of public health expenditures, but that of females was not affected by any expenditure of divisions (Figure 1 and Supplementary Tables S2–S4). Suicide mortalities caused by romance for males + females were decreased by the total amount of public health and police expenditures but were increased by that of public works (Figure 1 and Supplementary Table S2). Suicide mortalities caused by romance for males were decreased and increased by the total amount of expenditures of police and public works, respectively (Figure 1 and Supplementary Table S3); however, suicide mortalities caused by romance for females were decreased and increased by the total amount of expenditures of public health and public works, respectively (Figure 1 and Supplementary Table S4). Suicide mortalities with school-related motives for males were decreased by the total amount of expenditure of public health (Figure 1 and Supplementary Table S3), whereas neither males + females nor females were affected by any expenditure of divisions (Figure 1 and Supplementary Tables S2 and S4).

### 3.2. Relationship between Regional Financial Expenditure Per Capita of Six Divisions and Suicide Mortalities Caused by Six Major Motives (Model-2)

**Figure 2:**
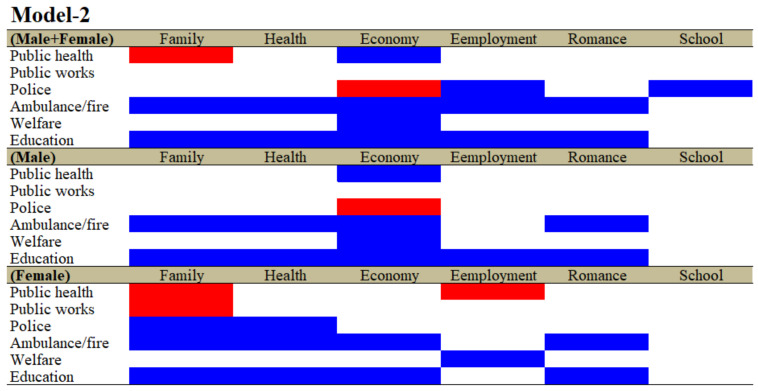
Relationship between financial expenditure per capita of six major divisions of prefectures/municipalities and suicide mortalities caused by six major motives for both genders (males + females), males and females between 2009 and 2018 using fixed- or random-effect analyses of hierarchal linear regression with robust standard error. Blue and red columns denote significant factors for decreasing and increasing suicide mortalities, respectively. The detailed statistical values are represented in Supplementary Tables S2–S4.

The hierarchical linear regression model also detected positive and negative relationships between the regional financial expenditure per capita of the six major divisions of prefectures/municipalities and the suicide mortalities caused by the six major suicide motives for males + females, males and females; however, the results of Model-1 and Model-2 were notably different. In particular, in Model-1, the regional financial expenditure per capita of ambulance/fire and education did not affect any suicide mortalities caused by the six major motives (Figure 1). However, expenditures per capita of both ambulance/fire and education contributed to reductions in suicide mortalities caused by motives associated with family, health, economy, employment and romance for males + females without affecting only those caused by school-related problems (Figure 2 and Supplementary Table S2). Expenditures per capita of both ambulance/fire and education contributed to reductions in suicide mortalities caused by motives associated with family, health, economy and romance for both males and females without affecting those caused by employment- or school-related problems (Figure 2 and Supplementary Tables S3 and S4). Contrarily, the relationships of expenditure per capita of welfare with suicide mortality were limited compared with those of ambulance/fire and education, since expenditure per capita of welfare provided reductions in suicide mortalities with economy-related motives for males + females and males, and employment motives for females (Figure 2 and Supplementary Tables S2–S4).

In Model-1, the expenditure of public works unexpectedly contributed to the increase in suicide mortalities caused by various motives, whereas the expenditures per capita of public works increased suicide mortality caused by family-related motives for females without affecting those caused by any motives for males + females and males (Figure 2 and Supplementary Tables S2–S4). Expenditures per capita of public health decreased suicide mortalities caused by economy-related motives for males + females and males but increased those caused by family- and employment-related motives for females (Figure 2 and Supplementary Tables S2–S4). The consistent relationship between expenditures per capita of police and suicide mortality could not be confirmed, since expenditures per capita of police increased the suicide mortalities caused by economy-related motives for males without affecting those of females, but decreased suicide mortalities caused by family- and health-related motives for females without affecting those of males (Figure 2 and Supplementary Tables S2–S4).

### 3.3. Relationship between Financial Expenditure Per Capita of Divisions and Subdivision of Education and Suicide Mortalities Caused by Motives (Model-3)

The deviation of the relationship between the expenditure of education and suicide mortalities between Model-1 and Model-2 suggests that the expenditure of education per capita plays an important role in suicide mortality. The SLPFS makes available the expenditures of six subdivisions of education per capita, namely, elementary school, junior high school, senior high school, special school, kindergarten and social education [46,47]. In Model-3, the replacement of education expenditure with the expenditures of the six education subdivisions elucidated the relationship between regional financial expenditures and suicide mortality, thereby allowing us to specifically understand the detailed effects of education expenditures on suicide mortality.

**Figure 3:**
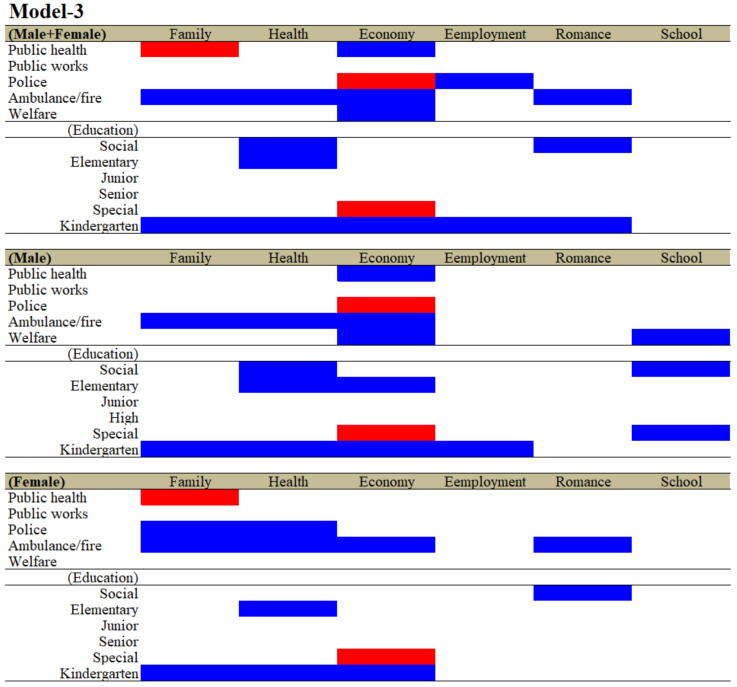
Relationship between financial expenditure per capita of divisions and subdivision of education of prefectures/municipalities, and suicide mortalities caused by six major motives for both genders (males + females), males and females from 2009 to 2018 using fixed- or random-effect analyses of hierarchal linear regression with robust standard error. Blue and red columns denote significant factors for decreasing and increasing suicide mortalities, respectively. The detailed statistical values are presented in Supplementary Tables S2–S4.

As a result of analysing education expenditures replaced by the six subdivisions, the relationship between the expenditures of the five other divisions and suicide mortalities disaggregated by the six motives was only slightly changed. In particular, the significantly negative relationships between the expenditures of police and ambulance/fire and suicide mortality of males + females caused by school and employment in Model-2 were abolished in Model-3, but other negative and positive relations remained to be observed (Figure 2 and Figure 3). The significantly negative relationships between the expenditures of ambulance/fire and suicide mortality of males caused by romance-related motives in Model-2 were abolished in Model-3 (Figure 2 and Figure 3). The significantly positive and negative relationships between the expenditures of public works and welfare and suicide mortalities of females caused by family and employment in Model-2, respectively, were also abolished in Model-3 (Figure 2 and Figure 3).

Regarding the relationships between the expenditures of education subdivisions and suicide mortalities, it was detected that increased kindergarten expenditures contributed to a decrease in suicide mortalities caused by a wide spectrum of motives. Other notable results were listed. Neither the expenditures of junior nor senior high schools affected suicide mortalities caused by any motives. Contrarily, the expenditure of special schools was observed to be positively related to suicide mortalities caused by the economy for males + females, males and females. Suicide mortality for males caused by school-related motives was reduced by increasing the expenditures of welfare, social education and special schools.

### 3.4. Relationship between Financial Expenditure Per Capita of Divisions and Subdivisions of Education and Welfare, and Suicide Mortalities Caused by Motives (Model-4)

The deviation associated with the relationship between the expenditure of welfare and suicide mortalities between Model-2 and Model-3 emphasised the notion that expenditures of kindergarten school probably play an important role in suicide mortality. That is, support for bringing up children may contribute to an improvement in suicide mortality. The SLPFS also makes available expenditure of four subdivisions of welfare per capita, namely, social welfare, elderly welfare, child welfare and livelihood [46,47]. In Model-4, the replacement of welfare expenditure with the expenditures of four welfare subdivisions allowed us to elucidate the relationship between the regional financial expenditures of these four subdivisions of welfare and suicide mortality.

**Figure 4:**
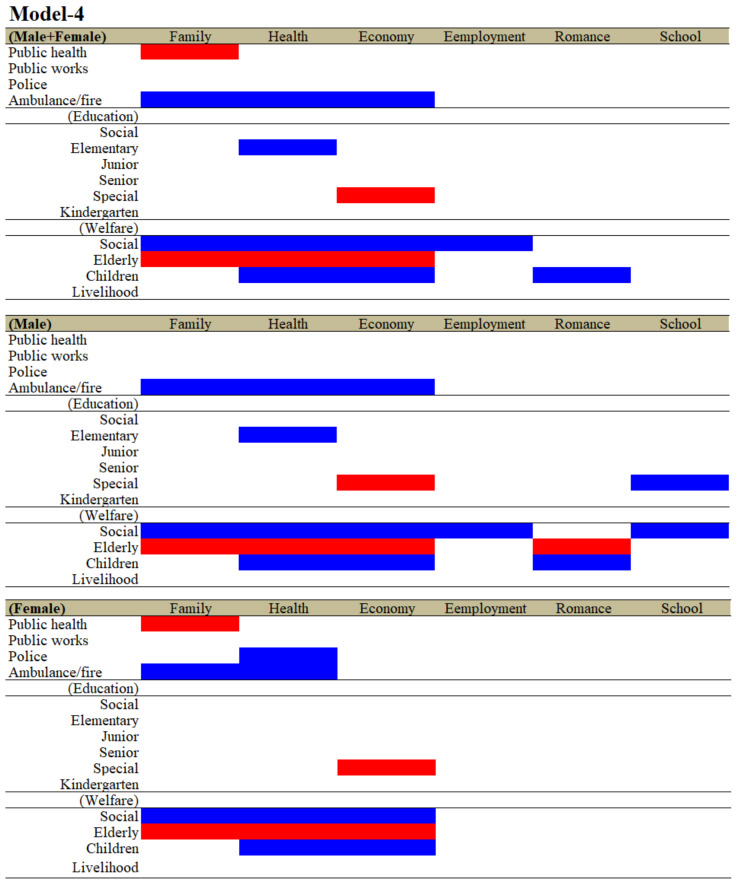
Relationship between financial expenditure per capita of divisions and subdivision of education and welfare of prefectures/municipalities, and suicide mortalities caused by six major motives for both genders (males + females), males and females from 2009 to 2018 using fixed- or random-effect analyses of hierarchal linear regression with robust standard error. Blue and red columns denote significant factors for decreasing and increasing suicide mortalities, respectively. The detailed statistical values are presented in Supplementary Tables S2–S4.

As a result of analysing welfare expenditures replaced by the four subdivisions, the relationships between the expenditures of the four divisions, except for education or welfare, and suicide mortalities caused by motives were drastically changed compared to those in Model-3. The significantly positive and negative relationships between the expenditures of police and suicide mortality of males + females caused by economy and employment in Model-3, respectively, were abolished in Model-4 (Figure 3 and Figure 4). The significantly negative relationship between the expenditures of public health and suicide mortality of males + females caused by economy-related motives in Model-3 was also abolished in Model-4 (Figure 3 and Figure 4). The significantly negative and positive relationships between the respective expenditures of public health and police and suicide mortalities of males caused by economy-related motives in Model-3 were also abolished in Model-4 (Figure 3 and Figure 4). The significantly negative relationships between the expenditures of police and suicide mortality of females caused by family-related problems in Model-3 were abolished in Model-4 (Figure 3 and Figure 4). The significantly negative relationships between the expenditures of ambulance/fire and suicide mortality of females caused by the economy and romance in Model-3 were also abolished in Model-4 (Figure 3 and Figure 4). Regarding the relationships between the expenditures of education subdivisions and suicide mortalities, the wide-spectrum, negative effects of kindergarten expenditures were surprisingly abolished in Model-4; however, the positive relationships between the expenditure of special school and suicide mortalities of males + females, males and females remained to be detected in Model-4.

**Figure 5:**
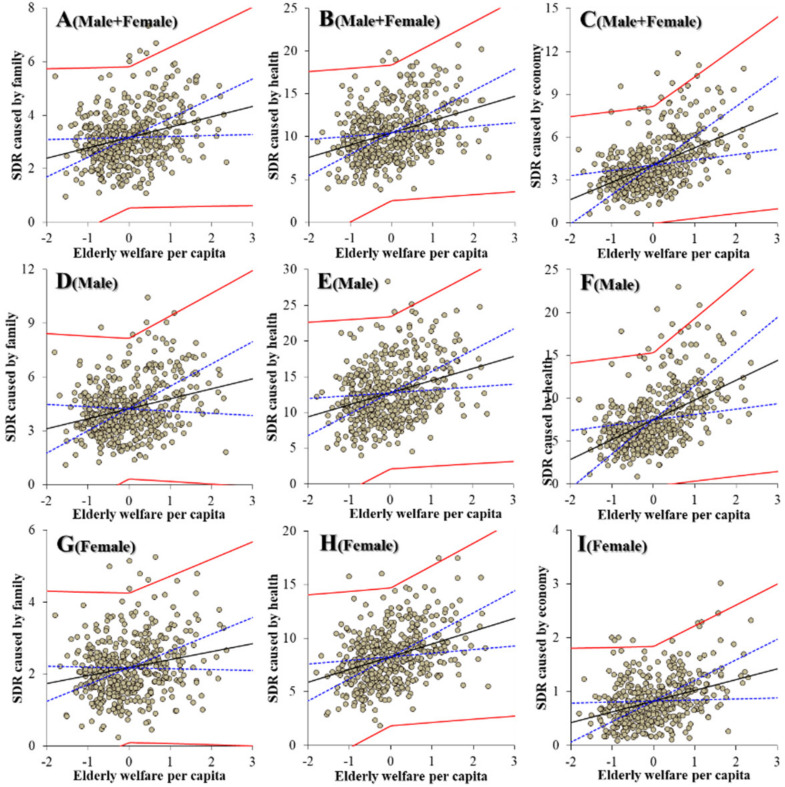
Relationship between financial expenditure per capita of elderly welfare of prefectures and municipalities, and suicide mortalities of males + females (**A**–**C**), males (**D**–**F**) and females (**G**–**I**) caused by motives associated with family (**A**,**D**,**G**), health (**B**,**E**,**H**) and economy (**C**,**F**,**I**), and suicide mortality of both genders (males + females) caused by family (**A**,**D**,**G**), health (**B**,**E**,**H**) and economy (**C**,**F**,**I**) from 2009 to 2018 by fixed- or random-effect analysis of hierarchal linear regression with robust standard error. Ordinates and abscissas indicate SDR of suicide mortalities (per 100,000 population) and centralised (by average) social welfare expenditure per capita of prefectures plus municipalities (JPY 10,000), respectively. Black, blue and red lines are the slope of fixed effects, standard deviation of fixed effects and fixed effects with random effects, respectively.

**Figure 6:**
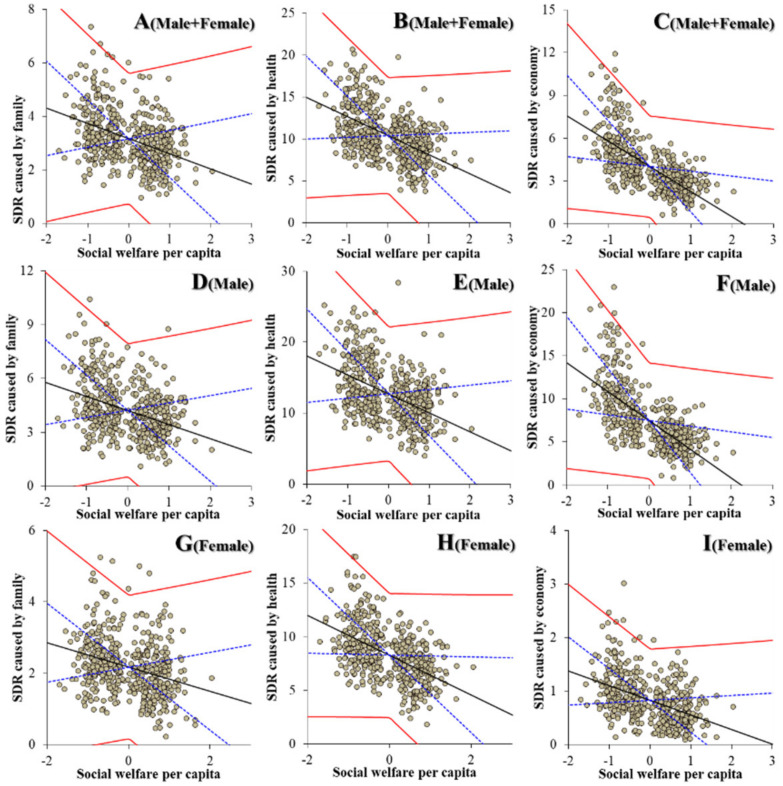
Relationship between financial expenditure per capita of social welfare of prefectures and municipalities, and suicide mortalities of males + females (**A**–**C**), males (**D**–**F**) and females (**G**–**I**) caused by motives associated with family (**A**,**D**,**G**), health (**B**,**E**,**H**) and economy (**C**,**F**,**I**), and suicide mortality of both genders (males + females) caused by family (**A**,**D**,**G**), health (**B**,**E**,**H**) and economy (**C**,**F**,**I**) from 2009 to 2018 by fixed- or random-effect analysis of hierarchal linear regression with robust standard error. Ordinates and abscissas indicate SDR of suicide mortalities (per 100,000 population) and centralised (by average) social welfare expenditure per capita of prefectures plus municipalities (JPY 10,000), respectively. Black, blue and red lines are the slope of fixed effects, standard deviation of fixed effects and fixed effects with random effects, respectively.

**Figure 7:**
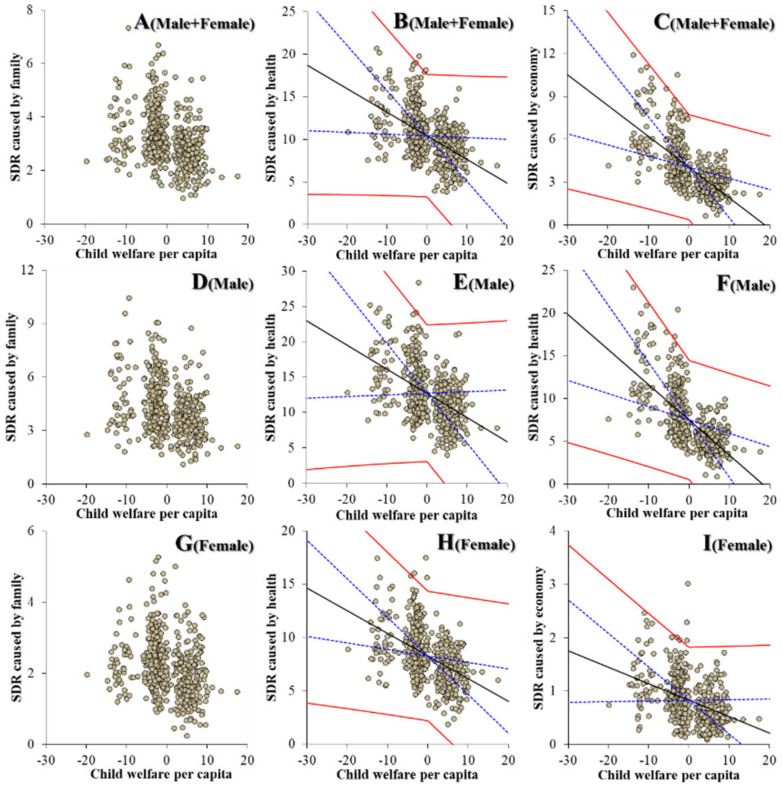
Relationship between financial expenditure per capita on child welfare of prefectures and municipalities, and suicide mortalities of males + females (**A**–**C**), males (**D**–**F**) and females (**G**–**I**) caused by motives associated with family (**A**,**D**,**G**), health (**B**,**E**,**H**) and economy (**C**,**F**,**I**), and suicide mortality of both genders (males + females) caused by family (**A**,**D**,**G**), health (**B**,**E**,**H**) and economy (**C**,**F**,**I**) from 2009 to 2018 using fixed- or random-effect analysis of hierarchal linear regression with robust standard error. Ordinates and abscissas indicate SDR of suicide mortalities (per 100,000 population) and centralised (by average) social welfare expenditure per capita of prefectures plus municipalities (JPY 10,000), respectively. Black, blue and red lines are the slope of fixed effects, standard deviation of fixed effects and fixed effects with random effects, respectively.

Contrary to the education subdivision, regarding the relationships between the expenditures of welfare subdivisions and suicide mortalities could be detected as a defined polarisation structure. Notably, the expenditures of elderly welfare contributed to an increase in suicide mortalities of males caused by motives associated with family, health, economy and romance (Figure 5). The expenditures of elderly welfare were also observed to be positively related to suicide mortalities of males + females and females caused by motives associated with family, health and economy (Figure 5). The expenditures of social welfare contributed to a decrease in suicide mortalities of males + females caused by motives associated with family, health, economy and employment. The expenditures of social welfare contributed to a decrease in suicide mortalities of males caused by motives associated with family, health, economy, employment and school (Figure 6). The expenditures of social welfare contributed to a decrease in suicide mortalities of females caused by motives associated with family, health and economy (Figure 6). The expenditures of child welfare contributed to a decrease in suicide mortalities of males + females and males caused by motives associated with health and romance (Figure 7). The expenditures of child welfare contributed to a decrease in suicide mortalities of females caused by motives associated with family and health (Figure 7).

### 3.5. Trends of Regional Financial Expenditure of Welfare Subdivision in Japan between 2009 and 2018

**Figure 8:**
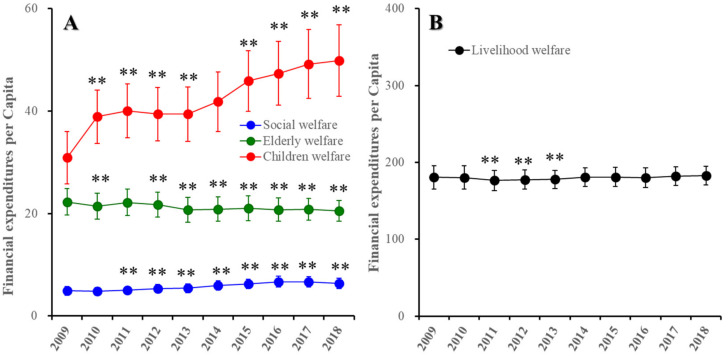
Trends of regional financial expenditures per capita of welfare subdivisions of social, elderly and child (**A**) and livelihood welfare (**B**) in Japan from 2009 to 2018. Ordinates indicate regional financial expenditure of prefecture and municipality (JPY 10,000 per capita). ** *p* < 0.01, relative to 2009 by linear mixed-effect model with Sidak’s post hoc test.

The annual expenditure of national government remained almost unchanged in the period of 2009–2018, but regional financial expenditure was increased by 18% from 2009 to 2018 [46,47,56]. From 2014 to 2018, the total amount of the regional financial expenditure of prefectures and their municipalities slightly but significantly increased compared to that in 2009 (18% increase in 2018 compared to 2009), whereas from 2013 to 2018, the regional financial expenditure of welfare division increased compared to that in 2009 (33% increase in 2018 compared to 2009) (data not shown) [35]. The expenditures per capita in 2009–2018 of social welfare (F (3.4, 157.4) = 451.1 (*p* < 0.01)) and child welfare (F (2.7, 95.3) = 634.8 (*p* < 0.01)) were increased compared to those in 2009, whereas the elderly expenditure per capita in 2009–2018 was decreased compared to that in 2009 (F (3.9, 177.8) = 69.3 (*p* < 0.01)) (Figure 8A). The expenditure of livelihood welfare per capita in 2009–2018 temporarily decreased from 2011 to 2013 compared to that in 2009 (F (2.2, 101.9) =1 5.0 (*p* < 0.01)) (Figure 8B).

## 4. Discussion

Suicide mortality in Japan, which maintained a high level of over 20 deaths per 100,000 population during the period of 1998–2009 (at maximum, 27.0 deaths per 100,000 population in 2003), began to reduce in 2009 and has been steadily decreasing since then (16.0 deaths per 100,000 population in 2019) [7,8,9,16]. There are some recent reports suggesting that Japanese governmental financial policies may play an important role as one of the factors contributing to the improvement in suicide mortality [7,15,16,34,35,57]. It is well known that appropriate or inappropriate governmental policies can suppress or accelerate the adverse reactions induced by various crises [57,58]. Historically, the expenditures of public works in New Deal relief after the Great Depression between 1929 and 1940 were negatively related to suicide mortality [57]. The adverse impact of the recessions on suicide mortality was weaker in countries with relatively larger social welfare expenditures [59,60,61], whereas austerity after the 2008 global financial crisis played an important role in increasing suicide mortality due to the deterioration of welfare in several countries, such as the U.K., Greece, Ireland, Portugal and Spain [20,22,23,24,25,26,27,28,29]. Regarding the Japanese financial situation, during the period of 2009–2018, annual national governmental expenditure remained almost unchanged, but regional financial expenditure was increased [46,47,56]. The influence of prioritising financial expenditures of regional governments in Japan during the period of 2009–2018 on suicide mortality has previously been analysed [35]. Between 2009 and 2018, only welfare expenditure was significantly increased in the regional governmental expenditures of the six divisions; however, the statistical analyses could not confirm that the reduction in suicide mortalities in Japan was affected by the total amount of welfare expenditure or welfare expenditure per capita [35]. Contrarily, in subdivided analyses of welfare finance, the expenditures of child and social welfares contributed to reductions in suicide mortality, but conversely, the expenditures of elderly welfare contributed to an increase in suicide mortality [35]. Furthermore, the series of analyses suggest that the governmental finances could contribute to a reduction in suicide mortality by protectively compensating the target problems of individuals associated with life-cycles and/or life–work–family balance [7,15,16,34,35,53]. Against this backdrop, in the present study, we determined the relationship between regional governmental expenditures and suicide mortalities caused by the six major suicidal motives.

In the analyses of the effects of the ratio of expenditures of the six divisions (Model-1), the expenditures of public health, public works and police affected a wide spectrum of suicide mortalities caused by suicidal motives, such as family, health, economy and romance and school problems. The ratios of both the expenditures of public health and police were observed to be negatively related to suicide mortality, whereas the ratio of the expenditure of public works was positively related to suicide mortality. Contrarily, in the analyses of the effects of expenditures per capita of the six divisions (Model-2), the expenditures per capita of ambulance/fire and education displayed a wide-spectrum, negative relationship with suicide mortalities caused by suicidal motives, such as family, health, economy, employment and romance problems (Model-2). Notably, the expenditure per capita of kindergarten predominantly reduced suicide mortalities compared to the expenditures in the other divisions and education subdivisions (Model-3). In Model-1, Model-2 and Model-3, the effects of welfare expenditures were limited and inconsistent, since the significantly negative relationships between welfare expenditure and suicide mortality in Models-1 were caused by family-related motives; in Model-2, they were caused by economy and employment; and in Model-3, they were caused by economy and notably school for males. Despite the limited inhibitory effects of expenditures of welfare, in the analyses of the effect of subdivided welfare expenditures in Model-4, the expenditures of the three subdivisions of welfare, namely, social, elderly and child welfare, contributed to suicide mortalities caused by a wide spectrum of suicidal motives and additionally neutralised the relationships between the expenditures of divisions, except for ambulance/fire. Therefore, these results indicate the possibility that each welfare subdivision may affect/compensate specific suicide motives, resulting in the prevention of suicidal behaviours. 

The increase in regional governmental expenditures per capita in Japan was related to a decrease in suicide mortalities of both males and females aged between 40 and 64 years [62]. It is well known that as a response to the most fundamental Japanese socioeconomic issues, namely, the declining birth rate and ageing population, the expenditure per capita on child welfare has been increasing. The expenditure on child welfare was increased by the revision of the Child Welfare Act in 2010 (to strengthen the cooperation between child welfare and education) [63] and the Outline of Measures for Society with Decreasing Birth Rate in 2015 [64]. A recent study reported that child welfare expenditures contributed reduced suicide mortalities in a wide range of age populations, whereas elderly welfare expenditure was observed to be positively related to suicide mortalities of working-age populations [35]. In Model-3, an increase in the expenditures of kindergarten was related to a decrease in suicide mortalities caused by a wide spectrum of suicidal motives, but the protective effect of kindergarten expenditure was abolished by the addition of expenditures of welfare subdivisions in Model-4. The results in Model-3 and Model-4 suggest that the prioritisation of child welfare expenditure probably contributed to maintaining the reducing trend of suicide mortality in Japan. This interaction between the expenditures of kindergarten education and child welfare suggests that support for childcare plays an important role in supporting livelihoods, but it also indicates that economic support is more fundamental than support of facilities and time. Although the total amount of expenditure of elderly welfare is increasing, the elderly population is also predominantly increasing compared to the increasing expenditure of elderly welfare, and this resulted in the decreased elderly welfare expenditure per capita between 2009 and 2018 [65]. 

In the present study, in Model-4, an increase in elderly welfare expenditure was related to an increase in suicide mortalities caused by family-, health- and economy-related motives for both males and females, whereas child welfare expenditures provided a reduction in suicide mortalities caused by health- and economy-related motives without affecting those caused by family-related motives for males and females. This governmental financial disadvantage of the elderly population is considered to be tolerated by the fact that the elderly population is economically affluent compared to the working-age population [66]. In addition, the majority of elderly population want to participate in society to make up for the decline in the working population [65]. Between 2009 and 2018, the shift in governmental welfare policy was a response to socioeconomic issues, the declining birth rate and the ageing population in Japan, but it likely also provided a reduction in suicide mortality as a by-product. 

Although the present study demonstrated the relationship between regional financial expenditures and suicide mortalities caused by six major suicidal motives in Japan, there are limitations to this work. Livelihood protection in Japan, which supports the minimum necessary living of persons and/or households due to disabilities, care for the aged, pregnancy or unemployment, comprises various assistances, such as medicine, education and housing assistances. It is possible that the present study could not detect any significant relationships due to the various forms of assistance provided to a wide range of residents. Notably, the design of this study attempted to identify the major expenditures by subdividing the expenditure divisions, contrary to general panel data analysis studies. However, the most serious limitation is that only one type of predictor (regional governmental expenditures) was analysed to explain a highly complex variable (suicide mortality), thus not identifying the effects of other psychosocial and/or socioeconomic factors. Therefore, to identify the actual sociopsychological pathomechanisms of suicide, more detailed regional financial expenditures and psychosocial factors should be restructured according to the target, and their relationships with suicide mortality caused by suicidal motives disaggregated by gender and ages should be analysed.

## 5. Conclusions

The present study demonstrates the importance of the association between regional financial expenditures and suicide mortality caused by six major suicidal motives (family-, health-, economy-, employment-, romance- and school-related motives) between 2009 and 2018 in Japan, using a fixed-effect analysis of hierarchal linear regression with robust standard error. The expenditures of ambulance/fire services, welfare and education played important roles in the reduction in suicide mortality, at least in some statistical indicators; however, the expenditure of public works displayed no relationship with male suicide mortality but instead may have increased female suicide mortality caused by family-related motives. The most effective expenditure in the six major divisions was that of welfare. In the welfare expenditure subdivisions, child and social welfare expenditures were effective in achieving a reduction in suicide mortality, but elderly welfare expenditure contributed to an increase in suicide mortality. The child and social welfare expenditures contributed to reductions in a wide range of suicide mortalities of males and females, such as those caused by family-, health- and economy-related problems, whereas the elderly welfare expenditure conversely increased them. It is difficult to drastically revise the expenditures due to policies of regional governments to improve the declining birth rate and ageing population in Japan, since regional financial expenditure is fundamental for maintaining and operating welfare systems. Although the relatively increasing and decreasing expenditures of elderly and child welfare, respectively, are unavoidable due to the social concerns associated with the declining birth rate and ageing population in Japan, we must study the rational redistribution of welfare expenditure via the identification of socioeconomic and sociopsychological factors.

## Data Availability

All data relevant to the study are included in the article or uploaded as Supplementary Information. All raw data are available to any persons from Japanese National databases in the Statistics Bureau of the Ministry of Internal Affairs and Communications (SBMIAC), Cabinet Office (CAO) and Ministry of Health, Labour and Welfare (MHLW).

## Supplementary Materials

The following are available online at https://www.mdpi.com/article/10.3390/ijerph19010084/s1, Table S1: Feature of regional governmental expenditures, Table S2: Impacts of financial expenditure ratio (Model-1) and per Capita (Model-2,3,4) of divisions and subdivisions of prefectures and municipalities on suicide mortality of male plus female caused by major six motives, Table S3: Impacts of financial expenditure ratio (Model-1) and per Capita (Model-2,3,4) of divisions and subdivisions of prefectures and municipalities on suicide mortality of male caused by major six motives, Table S4: Impacts of financial expenditure ratio (Model-1) and per Capita (Model-2,3,4) of divisions and subdivisions of prefectures and municipalities on suicide mortality of female caused by major six motives.
