# Supplementary material for: Relationships between Expenditure of Regional Governments and Suicide Mortalities Caused by Six Major Motives in Japan
Source: Int J Environ Res Public Health. 2021 Dec 22;19(1):84. doi: 10.3390/ijerph19010084 (PMC8751063; doi:10.3390/ijerph19010084)
Supplement: Supplementary file 1 [file ijerph-19-00084-s001.zip › ijerph-1471603-supplementary.pdf]

Supplemental Data List

Supplementary Table S1: Feature of regional governmental expenditures. (page 2).

Supplementary Table S2: Impacts of financial expenditure ratio (Model-1) and per Capita (Model-2,3,4) of divisions and subdivisions of prefectures and municipalities on suicide mortality of male plus female caused by major six motives. (page 3).

Supplementary Table S3: Impacts of financial expenditure ratio (Model-1) and per Capita (Model-2,3,4) of divisions and subdivisions of prefectures and municipalities on suicide mortality of male caused by major six motives. (page 4).

Supplementary Table S4: Impacts of financial expenditure ratio (Model-1) and per Capita (Model-2,3,4) of divisions and subdivisions of prefectures and municipalities on suicide mortality of female caused by major six motives. (page 5).

Supplementary Table S1:

Feature of regional governmental expenditures

Division/Subdivision	Subject/Content
**public health**	public health expenditure of prefecture/municipality comprises support for or the improvement of medical care, mental health and collecting/disposing of general waste, to maintain and improve public health and the regional environment.
**public works**	public works expenditure of prefecture/municipality comprises constructing new public facilities and the improvement of existing ones, including roads, bridges, parks and sewers.
**police**	police expenditure comprises salaries of police officers, police construction, projects and traffic signals, to carry out police administration to prevent crime, ensure traffic safety, maintain the safety and order of regional communities, and protect the lives and property of individuals.
**Ambulance/fire**	Ambulance/fire services expenditure of municipality comprises salaries of fire-station officers, station construction and fire trucks and ambulances, to protect individuals from disasters for emergency cases and the sudden deterioration of health.
**Education**	Education expenditure of prefecture/municipality comprises salaries of educational officers, constructing new educational facilities and improving existing educational facilities.
**Social**	facilities including public halls, libraries and museums
Elementary school	6~12 years old
Junior high school	12~15 years old
Senior high school	15~18 years old
Special school	for the education of special needs individuals: prefecture: 3~18 years old
Kindergarten school	3~6 years old
**Welfare**	Welfare expenditure of prefecture/municipality comprises mainly social assistance expenditure.
Social	Various support for individuals with disabilities and welfare assistance for unclassifiable subjects.
Elderly	Financial support contributions for health insurance of elderly and long-term care insurance (for individuals 65 years old and over)
Children	Support for caregiver (excluding livelihood welfare recipients) raising child (up to 15 years old): allowance, school lunches and medical expenses subsidy (for individuals younger than 17 years old)
Livelihood	Support for livelihood-protection individuals

Supplementary Table S2:

Impacts of financial expenditure ratio (Model-1) and per Capita (Model-2,3,4) of divisions and subdivisions of prefectures and municipalities on suicide mortality of male plus female caused by major six motives.

Factors	Family				Health				Economy			
	β	SE	T	p	β	SE	T	p	β	SE	T	p
**Model-1**												
public health	-0.47	0.10	-3.70	0.001 **	-0.48	0.29	-4.26	0.000 **	-0.44	0.16	-4.12	0.000 **
public works	0.45	0.04	3.02	0.004 **	0.47	0.13	3.10	0.003 **	0.67	0.08	4.18	0.000 **
police	0.03	0.01	0.27	0.792	-0.28	0.02	-3.45	0.001 **	-0.34	0.02	-3.36	0.002 *
Ambulance/fire	-0.40	0.01	-1.18	0.245	-0.04	0.02	-0.10	0.919	0.44	0.02	1.00	0.322
Welfare	-0.11	0.01	-2.10	0.042 *	0.01	0.06	0.17	0.863	-0.19	0.06	-1.56	0.127
Education	0.05	0.05	1.00	0.325	0.01	0.14	0.29	0.773	0.00	0.08	-0.10	0.922
	(VC=0.254, X^2=295.08, p<0.01)				(VC=3.939, X^2=717.00, p<0.01)				(VC=0.465, X^2=249.47, p<0.01)			
**Model-2**												
public health	0.10	0.02	3.03	0.004 **	0.00	0.06	-0.05	0.963	-0.09	0.04	-2.70	0.010 *
public works	0.14	0.02	1.72	0.093	0.05	0.12	0.34	0.732	-0.04	0.12	-0.20	0.843
police	-0.43	0.53	-1.83	0.074	-0.22	1.40	-1.10	0.280	0.50	0.89	2.36	0.023 *
Ambulance/fire	-0.54	0.16	-5.46	0.000 **	-0.53	0.51	-5.22	0.000 **	-0.82	0.35	-7.01	0.000 **
Welfare	-0.10	0.01	-1.46	0.151	-0.15	0.08	-1.03	0.311	-0.44	0.07	-2.17	0.036 *
Education	-0.74	0.11	-3.27	0.002 **	-0.87	0.29	-4.51	0.000 **	-0.75	0.16	-4.30	0.000 **
	(VC=0.280, X^2=311.69, p<0.01)				(VC=3.872, X^2=527.56, p<0.01)				(VC=0.412, X^2=164.48, p<0.01)			
**Model-3**												
public health	0.10	0.02	2.85	0.007 **	0.00	0.06	-0.11	0.910	-0.09	0.04	-2.25	0.031 *
public works	0.11	0.02	1.69	0.100	0.00	0.10	0.02	0.986	-0.11	0.10	-0.57	0.574
police	-0.38	0.53	-1.61	0.116	-0.23	1.34	-1.23	0.228	0.46	0.82	2.35	0.024 *
Ambulance/fire	-0.54	0.16	-5.62	0.000 *	-0.52	0.46	-5.69	0.000 **	-0.79	0.29	-8.10	0.000 **
Welfare	-0.08	0.01	-1.38	0.176	-0.11	0.06	-0.95	0.350	-0.37	0.06	-2.19	0.035 *
(Education)												
Social	-0.05	0.20	-0.64	0.526	-0.16	0.49	-2.60	0.013 *	-0.15	0.37	-1.98	0.055
Elementary	-0.19	0.01	-1.30	0.201	-0.28	0.02	-2.46	0.019 *	-0.24	0.02	-1.99	0.055
Junior	0.01	0.01	0.07	0.946	0.01	0.04	0.08	0.936	-0.07	0.02	-0.56	0.579
High	-0.08	0.01	-0.83	0.409	-0.01	0.02	-0.07	0.943	0.07	0.01	0.85	0.402
Special	0.03	0.00	0.27	0.789	0.16	0.00	1.86	0.072	0.26	0.00	2.84	0.007 **
Kindergarten	-0.39	0.01	-3.64	0.001 **	-0.50	0.02	-3.58	0.001 **	-0.59	0.01	-5.97	0.000 **
	(VC=0.270, X^2=309.08, p<0.01)				(VC=3.955, X^2=593.532, p<0.01)				(VC=0.396, X^2=179.70, p<0.01)			
**Model-4**												
public health	0.11	0.02	3.02	0.005 **	0.03	0.07	0.68	0.498	-0.06	0.05	-1.32	0.196
public works	0.12	0.02	1.97	0.057	0.00	0.05	-0.03	0.980	-0.13	0.04	-1.83	0.077
police	-0.44	0.60	-1.68	0.102	-0.26	1.09	-1.67	0.105	0.30	0.81	1.58	0.124
Ambulance/fire	-0.34	0.18	-3.04	0.005 **	-0.23	0.39	-3.02	0.005 **	-0.48	0.23	-6.31	0.000 **
(Education)												
Social	0.05	0.16	0.79	0.437	-0.01	0.47	-0.21	0.832	0.02	0.30	0.38	0.704
Elementary	-0.13	0.01	-0.89	0.380	-0.16	0.02	-2.20	0.035	-0.12	0.01	-1.10	0.277
Junior	0.03	0.01	0.22	0.830	0.09	0.02	1.06	0.296	-0.09	0.02	-0.95	0.347
High	-0.06	0.01	-0.66	0.513	0.08	0.01	1.25	0.219	0.11	0.01	1.85	0.073
Special	0.00	0.00	-0.02	0.982	0.08	0.00	1.61	0.118	0.20	0.00	2.85	0.007 **
Kindergarten	-0.13	0.01	-1.24	0.223	-0.04	0.01	-0.51	0.614	-0.05	0.01	-0.76	0.453
(Welfare)												
Social	-0.30	0.11	-2.38	0.023 *	-0.36	0.19	-5.27	0.000 **	-0.39	0.13	-4.76	0.000 **
Elderly	0.35	0.05	2.78	0.009 **	0.23	0.11	2.69	0.011 *	0.38	0.07	3.86	0.000 **
Children	0.00	0.01	0.00	0.998	-0.26	0.02	-4.29	0.000 **	-0.40	0.02	-5.46	0.000 **
Livelihood	0.01	0.01	0.09	0.930	-0.07	0.02	-0.84	0.408	0.10	0.02	0.86	0.395
	(VC=0.256, X^2=313.33, p<0.01)				(VC=4.462, X^2=866.14, p<0.01)				(VC=0.621, X^2=342.33, p<0.01)			

Factors	Employment				Romance				School			
	β	SE	T	p	β	SE	T	p	β	SE	T	p
**Model-1**												
public health	-0.35	0.07	-2.68	0.011 *	-0.23	0.03	-2.09	0.043 *	-0.12	0.02	-1.06	0.293
public works	0.30	0.03	1.86	0.070	0.43	0.02	2.11	0.041 *	0.03	0.01	0.20	0.846
police	-0.07	0.01	-0.63	0.529	-0.28	0.00	-2.72	0.010 **	-0.04	0.00	-0.45	0.658
Ambulance/fire	-0.01	0.00	-0.03	0.977	-0.03	0.00	-0.07	0.942	0.32	0.00	0.76	0.452
Welfare	0.01	0.02	0.11	0.913	0.02	0.01	0.31	0.755	0.13	0.00	1.73	0.091
Education	0.05	0.03	0.93	0.356	0.01	0.01	0.22	0.828	0.05	0.01	1.44	0.156
	(VC=0.086, X^2=247.75, p<0.01)				(VC=0.013, X^2=145.99, p<0.01)				(VC=0.004, X^2=134.89, p<0.01)			
**Model-2**												
public health	0.02	0.01	0.51	0.616	-0.03	0.01	-0.63	0.531	0.00	0.00	-0.11	0.912
public works	0.02	0.03	0.10	0.920	-0.09	0.01	-1.07	0.289	-0.13	0.00	-1.60	0.116
police	-0.56	0.25	-3.11	0.003 **	-0.29	0.16	-1.21	0.235	-0.63	0.10	-2.58	0.014 *
Ambulance/fire	-0.27	0.12	-2.24	0.030 *	-0.39	0.06	-3.12	0.003 **	0.00	0.03	0.03	0.979
Welfare	-0.10	0.01	-1.48	0.146	-0.14	0.01	-1.29	0.203	0.02	0.00	0.19	0.848
Education	-0.44	0.06	-2.06	0.046 *	-0.53	0.03	-2.92	0.006 **	-0.10	0.02	-0.47	0.640
	(VC=0.113, X^2=296.60, p<0.01)				(VC=0.012, X^2=134.14, p<0.01)				(VC=0.006, X^2=159.61, p<0.01)			
**Model-3**												
public health	0.02	0.02	0.53	0.601	-0.03	0.01	-0.72	0.475	-0.02	0.01	-0.28	0.778
public works	-0.03	0.02	-0.20	0.843	-0.12	0.01	-1.56	0.126	-0.14	0.00	-1.57	0.125
police	-0.55	0.25	-3.01	0.005 **	-0.26	0.17	-1.01	0.318	0.09	0.03	1.06	0.296
Ambulance/fire	-0.23	0.14	-1.65	0.108	-0.33	0.06	-2.71	0.010 *	0.04	0.03	0.37	0.714
Welfare	-0.06	0.01	-1.18	0.244	-0.11	0.00	-1.33	0.190	0.01	0.00	0.11	0.910
(Education)												
Social	-0.10	0.09	-1.88	0.069	-0.18	0.07	-2.16	0.037 *	-0.13	0.04	-1.37	0.180
Elementary	-0.01	0.01	-0.08	0.936	-0.17	0.00	-1.50	0.143	0.06	0.00	0.50	0.618
Junior	-0.05	0.01	-0.35	0.730	0.08	0.00	0.49	0.625	-0.01	0.00	-0.05	0.960
High	0.00	0.00	0.00	0.997	-0.05	0.00	-0.46	0.645	-0.02	0.00	-0.23	0.821
Special	0.17	0.00	1.49	0.146	0.12	0.00	1.11	0.273	-0.09	0.00	-1.03	0.309
Kindergarten	-0.35	0.00	-3.09	0.004 **	-0.31	0.00	-2.45	0.019 *	-0.20	0.00	-1.73	0.093
	(VC=0.101, X^2=281.44, p<0.01)				(VC=0.010, X^2=122.367, p<0.01)				(VC=0.004, X^2=134.52, p<0.01)			
**Model-4**												
public health	0.03	0.02	0.50	0.617	-0.02	0.01	-0.28	0.780	-0.02	0.00	-0.45	0.658
public works	-0.02	0.01	-0.32	0.748	-0.13	0.01	-1.48	0.148	-0.15	0.00	-1.74	0.091
police	-0.08	0.18	-0.57	0.570	-0.32	0.16	-1.31	0.200	0.22	0.06	1.50	0.142
Ambulance/fire	-0.05	0.11	-0.44	0.663	-0.16	0.06	-1.32	0.197	0.07	0.03	0.65	0.520
(Education)												
Social	-0.03	0.13	-0.41	0.682	-0.09	0.07	-1.03	0.312	-0.13	0.03	-1.71	0.097
Elementary	0.06	0.00	0.55	0.585	-0.08	0.00	-0.65	0.519	0.07	0.00	0.69	0.497
Junior	-0.04	0.01	-0.34	0.734	0.12	0.00	0.87	0.393	-0.01	0.00	-0.10	0.919
High	-0.02	0.00	-0.26	0.798	0.00	0.00	-0.02	0.986	-0.04	0.00	-0.43	0.671
Special	0.10	0.00	1.39	0.173	0.07	0.00	0.86	0.396	-0.10	0.00	-1.18	0.245
Kindergarten	-0.18	0.00	-1.60	0.119	0.02	0.00	0.15	0.880	-0.19	0.00	-1.33	0.192
(Welfare)												
Social	-0.26	0.06	-2.48	0.018 *	-0.11	0.03	-0.96	0.346	-0.03	0.02	-0.24	0.810
Elderly	0.19	0.04	1.35	0.187	0.20	0.02	1.22	0.231	0.02	0.01	0.10	0.922
Children	-0.03	0.01	-0.33	0.746	-0.30	0.00	-3.12	0.004 *	0.00	0.00	0.03	0.977
Livelihood	0.00	0.01	-0.03	0.978	-0.03	0.00	-0.22	0.825	0.06	0.00	0.31	0.756
	(VC=0.092, X^2=266.89, p<0.01)				(VC=0.009, X^2=123.03, p<0.01)				(VC=0.002, X^2=95.86, p<0.01)			

β: coefficient value, SE: standard error, VC: variance component, X^2: Chi-square value.

*p<0.05 and **p<0.01 by hierarchical linear regression model analysis.

Supplementary Table S3:
Impacts of financial expenditure ratio (Model-1) and per Capita (Model-2,3,4) of divisions and subdivisions of prefectures and municipalities on suicide mortality of male caused by major six motives.

Factors	Family				Health				Economy				Employment				Romance				School			
	β	SE	T	p	β	SE	T	p	β	SE	T	p	β	SE	T	p	β	SE	T	p	β	SE	T	p
**Model-1**																								
public health	-0.37	0.14	-3.25	0.002 **	-0.38	0.43	-2.97	0.005 **	-0.42	0.31	-3.85	0.000 **	-0.29	0.12	-2.37	0.022 *	-0.04	0.04	-0.29	0.770	-0.26	0.02	-3.04	0.004 *
public works	0.49	0.06	3.46	0.001 **	0.38	0.20	2.20	0.034 *	0.70	0.16	4.26	0.000 **	0.21	0.05	1.27	0.212	0.64	0.02	3.53	0.001 **	0.08	0.01	0.51	0.615
police	0.00	0.02	0.04	0.968	-0.31	0.04	-3.53	0.001 **	-0.34	0.04	-3.22	0.002 **	-0.11	0.01	-1.02	0.312	-0.27	0.00	-2.38	0.022 *	0.07	0.00	0.78	0.439
Ambulance/fire	-0.41	0.01	-1.32	0.194	0.11	0.04	0.23	0.817	0.39	0.03	0.88	0.386	-0.24	0.01	-0.74	0.463	-0.67	0.00	-1.98	0.054	0.01	0.00	0.03	0.978
Welfare	-0.14	0.02	-2.36	0.023 *	0.00	0.08	-0.04	0.965	-0.22	0.11	-1.71	0.094	-0.14	0.04	-1.10	0.277	0.01	0.02	0.08	0.937	0.03	0.01	0.26	0.795
Education	0.00	0.05	-0.09	0.930	-0.01	0.18	-0.30	0.768	-0.02	0.16	-0.41	0.685	-0.03	0.05	-0.60	0.550	0.01	0.02	0.18	0.858	0.08	0.02	1.04	0.303
	(VC=0.472, X^2=241.21, p<0.01)				(VC=6.944, X^2=654.93, p<0.01)				(VC=1.605, X^2=240.02, p<0.01)				(VC=0.309, X^2=236.39, p<0.01)				(VC=0.023, X^2=115.92, p<0.01)				(VC=0.012, X^2=125.75, p<0.01)			
**Model-2**																								
public health	0.03	0.02	1.05	0.298	-0.03	0.09	-0.67	0.509	-0.11	0.07	-3.02	0.004 **	-0.01	0.02	-0.18	0.859	-0.02	0.02	-0.28	0.782	0.04	0.01	0.60	0.549
public works	0.10	0.03	1.19	0.243	0.04	0.15	0.32	0.751	-0.05	0.22	-0.23	0.823	0.09	0.06	0.52	0.606	-0.12	0.01	-1.10	0.276	0.03	0.01	0.27	0.787
police	-0.25	0.84	-1.02	0.312	-0.07	1.84	-0.35	0.726	0.50	1.60	2.50	0.017 *	-0.16	0.60	-0.72	0.478	-0.07	0.27	-0.27	0.792	-0.03	0.17	-0.11	0.910
Ambulance/fire	-0.50	0.22	-5.70	0.000 **	-0.43	0.54	-5.30	0.000 **	-0.83	0.68	-7.03	0.000 **	-0.15	0.15	-1.92	0.061	-0.20	0.06	-2.50	0.016 *	0.02	0.04	0.26	0.794
Welfare	-0.11	0.02	-1.77	0.083	-0.14	0.09	-1.12	0.271	-0.45	0.13	-2.19	0.034 *	-0.12	0.02	-1.60	0.117	-0.12	0.01	-1.43	0.161	-0.08	0.00	-1.13	0.265
Education	-0.78	0.18	-3.12	0.003 **	-0.88	0.44	-3.91	0.000 **	-0.77	0.31	-4.24	0.000 **	-0.68	0.18	-2.15	0.038 *	-0.50	0.04	-2.47	0.018 *	-0.12	0.03	-0.58	0.564
	(VC=0.504, X^2=250.65, p<0.01)				(VC=6.469, X^2=494.38, p<0.01)				(VC=1.404, X^2=159.93, p<0.01)				(VC=0.418, X^2=285.93, p<0.01)				(VC=0.019, X^2=100.72, p<0.01)				(VC=0.014, X^2=138.53, p<0.01)			
**Model-3**																								
public health	0.02	0.02	0.77	0.448	-0.03	0.09	-0.75	0.458	-0.10	0.08	-2.58	0.014 *	-0.01	0.02	-0.14	0.888	-0.02	0.02	-0.39	0.696	0.03	0.01	0.53	0.598
public works	0.07	0.03	1.01	0.319	-0.01	0.12	-0.08	0.940	-0.11	0.18	-0.60	0.553	0.04	0.05	0.28	0.778	-0.14	0.01	-1.23	0.225	0.05	0.01	0.32	0.749
police	-0.23	0.81	-0.96	0.342	-0.11	1.82	-0.57	0.573	0.46	1.50	2.45	0.019 *	-0.18	0.57	-0.85	0.403	-0.03	0.30	-0.12	0.909	0.12	0.17	0.49	0.627
Ambulance/fire	-0.53	0.24	-5.45	0.000 **	-0.42	0.56	-4.98	0.000 **	-0.80	0.57	-8.09	0.000 **	-0.13	0.19	-1.27	0.213	-0.14	0.09	-1.16	0.255	0.02	0.05	0.23	0.822
Welfare	-0.09	0.02	-1.64	0.111	-0.11	0.08	-1.02	0.315	-0.38	0.11	-2.21	0.034 *	-0.08	0.01	-1.34	0.190	-0.11	0.01	-1.32	0.197	-0.12	0.00	-2.04	0.049 *
(Education)																								
Social	-0.01	0.25	-0.23	0.817	-0.15	0.71	-2.29	0.028 *	-0.14	0.67	-1.99	0.054	-0.04	0.33	-0.43	0.672	-0.22	0.15	-1.73	0.092	-0.17	0.05	-2.52	0.016 *
Elementary	-0.15	0.02	-0.91	0.370	-0.27	0.03	-2.31	0.027 *	-0.26	0.03	-2.14	0.040 *	-0.11	0.01	-0.66	0.514	-0.02	0.01	-0.14	0.893	0.16	0.00	1.01	0.318
Junior	-0.12	0.02	-0.69	0.494	-0.03	0.05	-0.24	0.814	-0.06	0.04	-0.40	0.691	-0.06	0.02	-0.44	0.660	-0.05	0.01	-0.27	0.787	0.08	0.01	0.40	0.688
High	-0.04	0.01	-0.39	0.698	-0.02	0.02	-0.25	0.802	0.06	0.02	0.66	0.511	-0.07	0.01	-0.82	0.417	-0.08	0.00	-0.64	0.524	0.04	0.00	0.32	0.754
Special	0.02	0.00	0.20	0.843	0.17	0.00	1.97	0.056	0.25	0.00	2.76	0.009 **	0.18	0.00	1.51	0.140	0.08	0.00	0.51	0.611	-0.20	0.00	-2.32	0.026 *
Kindergarten	-0.41	0.01	-3.74	0.001 **	-0.45	0.03	-3.42	0.002 **	-0.59	0.02	-5.78	0.000 **	-0.39	0.01	-2.60	0.013 *	-0.17	0.00	-1.51	0.139	-0.09	0.00	-1.19	0.243
	(VC=0.504, X^2=253.52, p<0.01)				(VC=6.898, X^2=563.23, p<0.01)				(VC=1.380, X^2=177.02, p<0.01)				(VC=0.385, X^2=275.66, p<0.01)				(VC=0.017, X^2=97.36, p<0.01)				(VC=0.012, X^2=127.23, p<0.01)			
**Model-4**																								
public health	0.02	0.05	0.44	0.666	-0.01	0.10	-0.12	0.906	-0.07	0.09	-1.65	0.109	0.03	0.02	0.95	0.351	-0.05	0.01	-0.87	0.390	0.06	0.01	1.12	0.269
public works	0.08	0.03	1.06	0.296	-0.02	0.06	-0.37	0.711	-0.12	0.07	-1.79	0.083	0.06	0.05	0.41	0.685	-0.13	0.01	-1.36	0.183	0.07	0.01	0.53	0.602
police	-0.32	0.72	-1.53	0.136	-0.16	1.53	-0.99	0.331	0.35	1.54	1.81	0.079	-0.09	0.65	-0.37	0.715	-0.25	0.30	-0.84	0.410	0.26	0.18	0.98	0.333
Ambulance/fire	-0.34	0.25	-3.27	0.002 **	-0.18	0.55	-2.15	0.039 *	-0.51	0.42	-7.05	0.000 **	0.02	0.20	0.24	0.815	0.06	0.08	0.51	0.614	0.16	0.05	1.57	0.126
(Education)																								
Social	0.07	0.31	0.91	0.368	-0.03	0.66	-0.47	0.638	0.03	0.63	0.48	0.633	0.06	0.26	0.69	0.497	-0.14	0.10	-1.61	0.117	-0.06	0.05	-0.92	0.364
Elementary	-0.08	0.01	-0.85	0.402	-0.16	0.02	-2.04	0.050 *	-0.13	0.03	-1.16	0.256	-0.06	0.01	-0.37	0.716	0.10	0.01	0.58	0.567	0.21	0.00	1.36	0.183
Junior	-0.11	0.02	-0.94	0.353	0.03	0.03	0.32	0.753	-0.07	0.03	-0.72	0.477	0.01	0.01	0.07	0.944	-0.05	0.01	-0.28	0.782	0.09	0.01	0.48	0.632
High	-0.03	0.01	-0.35	0.732	0.05	0.02	0.71	0.485	0.09	0.01	1.51	0.141	0.00	0.01	-0.02	0.986	-0.08	0.00	-0.73	0.473	0.09	0.00	0.77	0.447
Special	0.00	0.00	-0.07	0.947	0.11	0.00	1.93	0.063	0.14	0.00	2.22	0.033 *	0.13	0.00	1.23	0.226	0.03	0.00	0.23	0.823	-0.24	0.00	-2.78	0.009 **
Kindergarten	-0.15	0.01	-1.46	0.155	-0.03	0.02	-0.37	0.712	-0.09	0.01	-1.40	0.170	-0.14	0.01	-0.90	0.376	0.17	0.00	1.75	0.089	-0.10	0.00	-0.89	0.378
(Welfare)																								
Social	-0.25	0.13	-2.44	0.020 *	-0.27	0.26	-3.66	0.001 **	-0.37	0.27	-4.29	0.000 **	-0.32	0.14	-2.37	0.024 *	0.02	0.05	0.12	0.901	-0.35	0.03	-2.93	0.006 **
Elderly	0.36	0.08	2.68	0.011 *	0.21	0.15	2.27	0.030 *	0.37	0.15	3.69	0.001 **	-0.02	0.07	-0.15	0.880	0.62	0.04	3.10	0.004 **	0.06	0.02	0.41	0.682
Children	-0.03	0.02	-0.38	0.707	-0.29	0.03	-4.56	0.000 **	-0.40	0.03	-5.43	0.000 **	-0.09	0.02	-0.81	0.424	-0.26	0.01	-2.53	0.016 *	0.06	0.00	0.89	0.380
Livelihood	0.04	0.01	0.36	0.718	0.00	0.03	-0.01	0.989	0.08	0.03	0.71	0.483	-0.21	0.01	-1.38	0.177	-0.03	0.01	-0.16	0.874	-0.13	0.00	-1.04	0.307
	(VC=0.478, X^2=254.86, p<0.01)				(VC=8.111, X^2=800.89, p<0.01)				(VC=2.438, X^2=268.49, p<0.01)				(VC=0.320, X^2=248.06, p<0.01)				(VC=0.017, X^2=100.47, p<0.01)				(VC=0.006, X^2=90.76, p<0.01)			

β: coefficient value, SE: standard error, VC: variance component, X^2: Chi-square value.
*p<0.05 and **p<0.01 by hierarchal linear regression model analysis.

Supplementary Table S4:
Impacts of financial expenditure ratio (Model-1) and per Capita (Model-2,3,4) of divisions and subdivisions of prefectures and municipalities on suicide mortality of female caused by major six motives.

Factors	Family				Health				Economy				Employment				Romance				School			
	β	SE	T	p	β	SE	T	p	β	SE	T	p	β	SE	T	p	β	SE	T	p	β	SE	T	p
**Model-1**																								
public health	-0.47	0.09	-3.61	0.001**	-0.57	0.18	-6.45	0.000**	-0.48	0.04	-4.29	0.000**	-0.06	0.03	-0.48	0.634	-0.25	0.02	-2.51	0.016*	-0.13	0.01	-1.29	0.205
public works	0.24	0.04	1.47	0.150	0.55	0.10	3.99	0.000**	0.36	0.02	2.21	0.033*	0.22	0.01	1.17	0.248	0.42	0.01	2.85	0.007**	0.10	0.01	0.57	0.574
police	0.06	0.01	0.57	0.572	-0.18	0.02	-2.57	0.014*	-0.15	0.01	-1.33	0.190	0.10	0.00	0.79	0.434	-0.18	0.00	-1.59	0.119	0.08	0.00	0.90	0.373
Ambulance/fire	-0.27	0.01	-0.72	0.477	-0.27	0.01	-0.91	0.370	0.38	0.00	1.06	0.296	-0.24	0.00	-0.57	0.571	-0.03	0.00	-0.10	0.918	0.17	0.00	0.35	0.725
Welfare	-0.03	0.01	-0.37	0.717	0.04	0.04	0.54	0.592	0.05	0.01	0.46	0.644	-0.10	0.01	-0.79	0.436	0.08	0.01	0.97	0.340	0.03	0.00	0.31	0.759
Education	0.13	0.06	1.60	0.118	0.05	0.12	1.07	0.291	0.10	0.03	1.76	0.085	0.13	0.02	1.86	0.070	0.08	0.02	1.45	0.156	0.01	0.01	0.23	0.816
	(VC=0.120, X^2=179.14, p<0.01)				(VC=2.126, X^2=483.39, p<0.01)				(VC=0.016, X^2=101.78, p<0.01)				(VC=0.002, X^2=66.38, p<0.05)				(VC=0.008, X^2=95.73, p<0.01)				(VC=0.000, X^2=45.02, p>0.05)			
**Model-2**																								
public health	0.20	0.03	3.40	0.001**	0.04	0.05	1.17	0.250	0.03	0.01	1.01	0.320	0.12	0.00	4.38	0.000**	0.01	0.01	0.16	0.876	-0.01	0.00	-0.31	0.758
public works	0.16	0.02	2.35	0.024*	0.06	0.11	0.36	0.718	0.00	0.02	0.03	0.980	-0.12	0.00	-1.95	0.058	-0.01	0.01	-0.10	0.924	-0.10	0.00	-0.92	0.363
police	-0.58	0.39	-2.72	0.010*	-0.41	1.17	-2.03	0.049*	0.36	0.31	1.17	0.248	-0.35	0.16	-1.22	0.231	-0.24	0.19	-0.85	0.402	-0.01	0.10	-0.04	0.968
Ambulance/fire	-0.44	0.17	-3.33	0.002**	-0.60	0.56	-4.44	0.000**	-0.52	0.09	-4.37	0.000**	-0.12	0.05	-0.93	0.356	-0.26	0.06	-2.21	0.033*	-0.06	0.03	-0.49	0.625
Welfare	-0.05	0.01	-0.70	0.489	-0.13	0.07	-0.88	0.383	-0.23	0.01	-1.75	0.088	-0.12	0.00	-2.31	0.026*	-0.13	0.00	-1.48	0.145	-0.02	0.00	-0.50	0.617
Education	-0.44	0.07	-2.37	0.023*	-0.74	0.19	-4.70	0.000*	-0.38	0.04	-2.06	0.046*	0.11	0.03	0.46	0.648	-0.51	0.02	-3.26	0.002**	-0.17	0.01	-0.91	0.367
	(VC=0.137, X^2=195.92, p<0.01)				(VC=2.200, X^2=392.98, p<0.01)				(VC=0.017, X^2=95.68, p<0.01)				(VC=0.003, X^2=73.24, p<0.01)				(VC=0.006, X^2=81.21, p<0.01)				(VC=0.000, X^2=43.19, p>0.05)			
**Model-3**																								
public health	0.20	0.03	3.33	0.002**	0.04	0.05	1.14	0.260	0.03	0.01	0.90	0.372	0.12	0.01	1.64	0.109	0.00	0.01	-0.03	0.974	-0.01	0.00	-0.26	0.798
public works	0.15	0.02	1.91	0.064	0.02	0.09	0.13	0.896	-0.05	0.02	-0.30	0.764	-0.12	0.01	-1.26	0.216	-0.04	0.01	-0.36	0.721	-0.12	0.01	-1.11	0.272
police	-0.50	0.43	-2.12	0.041*	-0.39	1.08	-2.06	0.047*	0.33	0.29	1.18	0.245	-0.29	0.16	-1.04	0.305	-0.29	0.19	-1.05	0.300	-0.11	0.12	-0.33	0.741
Ambulance/fire	-0.40	0.15	-3.53	0.001**	-0.59	0.45	-5.40	0.000**	-0.45	0.09	-3.66	0.001**	-0.08	0.05	-0.60	0.555	-0.25	0.06	-2.09	0.044*	-0.06	0.03	-0.51	0.614
Welfare	-0.04	0.01	-0.59	0.558	-0.09	0.05	-0.81	0.422	-0.16	0.01	-1.64	0.109	-0.10	0.00	-1.24	0.222	-0.13	0.00	-1.71	0.095	-0.03	0.00	-0.80	0.430
(Education)																								
Social	-0.09	0.18	-1.04	0.305	-0.15	0.61	-1.63	0.111	-0.19	0.14	-1.55	0.130	0.00	0.07	-0.04	0.965	-0.23	0.07	-2.53	0.016*	-0.07	0.06	-0.55	0.582
Elementary	-0.21	0.01	-1.85	0.073	-0.25	0.02	-2.29	0.028*	-0.08	0.00	-0.57	0.570	0.10	0.00	0.76	0.453	-0.17	0.00	-1.27	0.211	-0.03	0.00	-0.21	0.836
Junior	0.23	0.01	1.72	0.094	0.07	0.03	0.54	0.596	-0.16	0.00	-1.39	0.174	-0.11	0.00	-0.71	0.485	0.01	0.00	0.03	0.975	0.07	0.00	0.50	0.621
High	-0.12	0.00	-1.33	0.192	0.02	0.01	0.22	0.830	0.12	0.00	1.51	0.141	0.00	0.00	-0.04	0.970	0.06	0.00	0.54	0.592	0.02	0.00	0.23	0.821
Special	0.03	0.00	0.40	0.694	0.12	0.00	1.40	0.170	0.26	0.00	2.84	0.007**	0.02	0.00	0.26	0.799	0.05	0.00	0.40	0.689	0.10	0.00	1.08	0.286
Kindergarten	-0.24	0.00	-2.22	0.033*	-0.51	0.02	-3.59	0.001**	-0.44	0.00	-4.49	0.000**	-0.04	0.00	-0.30	0.765	-0.17	0.00	-1.54	0.133	-0.01	0.00	-0.10	0.925
	(VC=0.125, X^2=187.32, p<0.01)				(VC=2.155, X^2=420.89, p<0.01)				(VC=0.013, X^2=89.04, p<0.01)				(VC=0.003, X^2=68.39, p<0.01)				(VC=0.003, X^2=63.96, p<0.01)				(VC=0.000, X^2=39.00, p>0.05)			
**Model-4**																								
public health	0.18	0.03	3.11	0.004**	0.06	0.04	1.97	0.057	0.05	0.01	1.10	0.281	0.04	0.01	0.82	0.417	0.01	0.01	0.34	0.733	-0.02	0.00	-0.76	0.453
public works	0.13	0.02	1.86	0.072	0.03	0.07	0.30	0.767	-0.05	0.01	-0.52	0.607	-0.11	0.00	-1.59	0.121	-0.03	0.01	-0.31	0.758	-0.14	0.01	-1.14	0.264
police	-0.05	0.18	-0.50	0.623	-0.38	0.99	-2.16	0.038*	0.23	0.29	0.79	0.433	-0.35	0.17	-1.18	0.248	-0.34	0.18	-1.29	0.205	-0.24	0.11	-0.83	0.415
Ambulance/fire	-0.18	0.12	-2.06	0.047*	-0.29	0.34	-3.52	0.001**	-0.23	0.11	-1.55	0.130	-0.06	0.05	-0.45	0.657	-0.07	0.07	-0.46	0.649	0.00	0.03	0.01	0.989
(Education)																								
Social	0.00	0.15	-0.02	0.981	0.00	0.38	0.08	0.936	-0.07	0.10	-0.89	0.382	0.00	0.06	0.02	0.986	-0.13	0.06	-1.76	0.088	-0.07	0.06	-0.54	0.590
Elementary	-0.07	0.01	-0.75	0.459	-0.15	0.02	-1.36	0.182	0.01	0.00	0.08	0.935	0.11	0.00	0.94	0.354	-0.08	0.00	-0.57	0.571	-0.02	0.00	-0.16	0.871
Junior	0.13	0.01	1.17	0.251	0.17	0.03	1.47	0.151	-0.15	0.00	-1.23	0.227	-0.17	0.00	-0.94	0.356	0.04	0.00	0.24	0.816	0.01	0.00	0.09	0.926
High	-0.10	0.00	-1.34	0.188	0.10	0.01	1.43	0.161	0.15	0.00	1.86	0.072	-0.04	0.00	-0.44	0.661	0.10	0.00	0.93	0.361	-0.02	0.00	-0.17	0.863
Special	-0.03	0.00	-0.50	0.619	0.04	0.00	0.52	0.609	0.22	0.00	2.71	0.011*	0.03	0.00	0.32	0.748	0.00	0.00	0.01	0.994	0.12	0.00	1.31	0.198
Kindergarten	-0.02	0.00	-0.28	0.778	-0.06	0.02	-0.42	0.675	-0.08	0.00	-0.69	0.496	-0.04	0.00	-0.19	0.849	0.15	0.00	1.14	0.263	0.02	0.00	0.11	0.914
(Welfare)																								
Social	-0.32	0.06	-3.81	0.001**	-0.44	0.16	-6.21	0.000**	-0.28	0.04	-3.07	0.004**	0.00	0.03	-0.03	0.974	-0.20	0.04	-1.54	0.132	-0.05	0.02	-0.44	0.666
Elderly	0.18	0.03	2.10	0.044*	0.25	0.10	2.57	0.015*	0.35	0.02	3.86	0.001**	0.23	0.02	1.22	0.231	0.25	0.02	1.45	0.156	0.27	0.01	1.30	0.201
Children	0.02	0.01	0.26	0.794	-0.16	0.02	-2.22	0.034*	-0.17	0.00	-1.99	0.055	0.05	0.00	0.62	0.539	-0.21	0.00	-2.02	0.051	0.07	0.00	0.91	0.368
Livelihood	-0.04	0.00	-0.50	0.617	-0.15	0.02	-1.21	0.236	0.05	0.00	0.59	0.561	-0.02	0.00	-0.11	0.913	-0.10	0.00	-0.60	0.551	0.27	0.00	1.24	0.224
	(VC=0.154, X^2=222.44, p<0.01)				(VC=2.294, X^2=551.16, p<0.01)				(VC=0.014, X^2=94.57, p<0.01)				(VC=0.002, X^2=65.21, p<0.01)				(VC=0.002, X^2=56.93, p<0.01)				(VC=0.000, X^2=27.72, p>0.05)			

β: coefficient value, SE: standard error, VC: variance component, X^2: Chi-square value.
*p<0.05 and **p<0.01 by hierarchal linear regression model analysis.
